# Intermuscular Coherence during Quiet Standing in Sub-Acute Patients after Stroke: An Exploratory Study

**DOI:** 10.3390/brainsci13121640

**Published:** 2023-11-26

**Authors:** Eiji Yamanaka, Ryosuke Goto, Michiyuki Kawakami, Takaki Tateishi, Kunitsugu Kondo, Ippei Nojima

**Affiliations:** 1 Department of Health Sciences, Graduate School of Medicine, Shinshu University, 3-1-1 Asahi, Matsumoto 390-8621, Japan; 21hm204k@shinshu-u.ac.jp; 2 Department of Rehabilitation Medicine, Tokyo Bay Rehabilitation Hospital, 4-1-1 Yatsu, Narashino 275-0026, Japan; 3 Department of Rehabilitation Medicine, Keio University School of Medicine, 35 Shinano-machi, Shinjuku-ku, Tokyo 160-8582, Japan; 4 Department of Rehabilitation Medicine, Nagoya City University, 1 Kawasumi, Mizuho-cho, Mizuho-ku, Nagoya 467-0001, Japan

**Keywords:** intermuscular coherence, EMG–EMG coherence, stroke, standing control, neuromuscular control, postural balance, postural sway, center of pressure

## Abstract

Asymmetrically impaired standing control is a prevalent disability among stroke patients; however, most of the neuromuscular characteristics are unclear. Therefore, the main purpose of this study was to investigate between-limb differences in intermuscular coherence during quiet standing. Consequently, 15 patients who had sub-acute stroke performed a quiet standing task without assistive devices, and electromyography was measured on the bilateral tibialis anterior (TA), soleus (SL), and medial gastrocnemius (MG). The intermuscular coherence of the unilateral synergistic (SL–MG) pair and unilateral antagonist (TA–SL and TA–MG) pairs in the delta (0–5 Hz) and beta (15–35 Hz) bands were calculated and compared between the paretic and non-paretic limbs. The unilateral synergistic SL–MG coherence in the beta band was significantly greater in the non-paretic limb than in the paretic limb (*p* = 0.017), while unilateral antagonist TA–MG coherence in the delta band was significantly greater in the paretic limb than in the non-paretic limb (*p* < 0.01). During quiet standing, stroke patients showed asymmetry in the cortical control of the plantar flexor muscles, and synchronous control between the antagonistic muscles was characteristic of the paretic limb. This study identified abnormal muscle activity patterns and asymmetrical cortical control underlying impaired standing balance in patients with sub-acute stroke using an intermuscular coherence analysis.

## 1. Introduction

Impaired standing control is a prevalent disability among patients with stroke [1]. It is conceivable that attaining early recuperation of the standing balance will result in the acquisition of a more utilitarian gait and ambulation ability in the community. In addition, fall-related incidents occurred primarily in inpatient rehabilitation wards and in the sub-acute post-stroke phase [2]. Thus, a proper assessment of standing and balance training is an essential component of early-stage stroke rehabilitation. In general, postural control in stroke patients with hemiparesis is characterized by impaired control in the paretic limb and compensation in the non-paretic limb [3], and a decreased contribution of the paretic limb to standing control has been reported to be associated with increased fall rates [4,5]. Consequently, within clinical and research contexts, it may be advantageous to evaluate the individual characteristics of each limb for the control of standing balance in stroke patients.

The assessment of postural control in stroke patients has predominantly been centered on the analysis of postural sway, which is achieved by quantifying the movement of the center of pressure (COP) during quiet standing using force plates. Previous studies have revealed that patients with stroke exhibit augmented COP sway and COP velocity, particularly in the anteroposterior and mediolateral directions, compared to healthy controls [6,7]. Furthermore, notable degrees of weight-bearing disparity and asymmetrical COP displacement in favor of the non-paretic side were observed in a substantial cohort of stroke patients [3,8]. These post-stroke deficits in postural control are believed to arise from the abnormal muscle activation patterns generated by the central nervous system (CNS). However, analysis of COP sway and weight-bearing asymmetry fails to provide insights into the intricate mechanisms governing neuromuscular regulation in the paretic limb and the compensatory mechanisms employed by the non-paretic limb. Therefore, it is important to delineate the neuromuscular control mechanisms underlying the foundational neural substrates governing perturbed COP dynamics during quiet standing in patients with stroke. Nevertheless, considerable ambiguity persists in our understanding of the characteristics of neuromuscular control during standing tasks in stroke patients.

Intermuscular coherence, a metric that quantifies the correlation between a pair of electromyographic (EMG) signals within each frequency band [9,10], has been the subject of scrutiny as an index of neuromuscular control during upright postural tasks. This phenomenon reflects the shared inputs of the descending neural drive among the motor neuron pools in the spinal cord. Consequently, intermuscular coherence offers valuable insight into the simultaneous control of multiple muscle combinations by the CNS. Furthermore, coherence within each frequency band is generated by distinct cerebral regions and neural circuits (spinal, subcortical, and cortical regions) [11]. Delta band coherence is indicative of descending inputs from subcortical sources and/or spinal circuits [12,13], whereas beta band coherence is related to the descending activity of the corticospinal tract [9,11,14]. Hence, intermuscular coherence provides a valuable estimate of the neural circuits and origins that actively contribute to motor tasks. 

Regarding a coherence analysis during quiet standing, healthy individuals demonstrated significant coherence between both bilateral and unilateral pairs of plantar flexors within the delta and certain alpha frequency bands [12,15,16]. Conversely, previous studies have reported that the coherence between antagonist muscles exhibits heightened values during more challenging standing tasks [15,17]. Consequently, it has been postulated that synchronously controlled muscle pairs could vary depending on the task complexity and inherent characteristics. Furthermore, the intermuscular coherence of the beta band, which reflects the activity in the corticospinal tract, was reported to increase with escalating difficulty in the standing task [16,18,19]. This observation may be attributed to voluntary and precise control of the functional muscle groups necessary for executing challenging tasks. A recent systematic scoping review [20] highlighted the investigation of intermuscular coherence as a tool to elucidate the intricacies of postural control in healthy individuals. However, its validation in individuals with CNS lesions, such as stroke, remains scarce. The exploration of intermuscular coherence in patients with stroke engaged in postural control is important considering that patients with stroke often exhibit abnormal muscle activation patterns [21,22,23] and compromised functionality of specific neuronal circuits, particularly the corticospinal tract on the lesion-affected side.

The primary objective of the present study was to examine neuromuscular regulation and delineate disparities between the paretic and non-paretic lower limbs during quiet standing control in patients with stroke using an analysis of intermuscular coherence. The present study introduced a novel approach by utilizing intermuscular coherence analysis to quantitatively assess asymmetries in the neuromuscular activation patterns and cortical muscle control. This sheds light on the mechanisms underpinning increased postural sway and weight-bearing asymmetry in stroke patients.

## 2. Materials and Methods

### 2.1. Study Design

The present study adopted an observational cross-sectional design involving sub-acute stroke patients undergoing inpatient rehabilitation. EMG data from the lower limbs during a quiet standing task were collected with the object of scrutinizing the intermuscular coherence disparities between the paretic and non-paretic limbs, as well as examining their associations with COP parameters and clinical assessment scales.

Our hypothesis posited that intermuscular coherence within the beta frequency band would manifest as a variation between the synergistic muscle pair in the paretic and non-paretic limbs, considering that patients with stroke often manifest asymmetries in the cortical control of muscles during functional tasks.

### 2.2. Participants

A total of 15 patients with sub-acute stroke were recruited for this study. Because of the novel nature of the study, we were unable to perform an a priori power analysis; thus, we chose this sample size based on previous studies examining the intermuscular coherence during gait tasks [24,25]. The participants were patients with stroke admitted to Tokyo Bay Rehabilitation Hospital, a convalescent rehabilitation facility located in Chiba, Japan. The inclusion criteria for the study were as follows: (1) patients who had experienced a stroke within the past 1–3 months; (2) patients exhibiting a supratentorial lesion in the unilateral cerebral hemisphere; and (3) patients who were able to maintain a standing posture for a minimum of one minute unaided by prosthetic devices, assistive apparatuses, or physical support. Individuals with other neurological, musculoskeletal, respiratory, or cardiovascular conditions that might potentially influence the postural control, as well as those who had experienced recurrent cerebrovascular incidents, were excluded from participation. Additionally, patients who were unable to understand the purpose and scope of the experiment or perform experimental tasks due to cognitive dysfunction, attention disorders, or aphasia were also excluded. All participants provided written informed consent prior to study initiation. This study was approved by the Institutional Review Boards of Tokyo Bay Rehabilitation Hospital (approval number: 272-2, approved 30 June 2021) and Shinshu University (approval number: 5760, approved 14 February 2023).

### 2.3. The Quiet Standing Task

**Figure 1:**
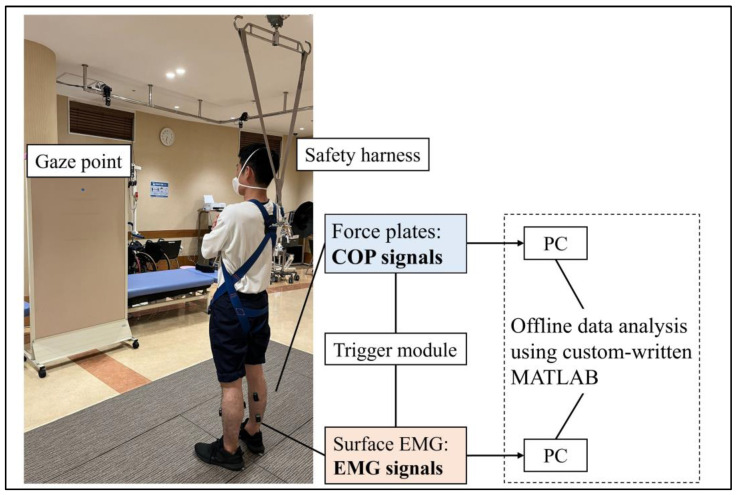
Representative summary of quiet standing task conditions.

Each participant performed a quiet standing task, with the weight bearing naturally determined by each individual. The task was performed in a bipedal stance with the participants’ arms crossed in front of their chest and both feet stationed 17 cm apart, pointing 14° outward from the sagittal plane [26]. The duration of the task was 40 s, and the participants were instructed to fix their gaze on a point two meters away. The use of any prosthetic device was prohibited during the execution of the standing task. Safety harnesses were worn to mitigate the risk of falling; however, weight support was disallowed. A physical therapist monitored the participants for safety during the tasks. The foot placement was marked using adhesive tape and the participants were mandated to uphold this arrangement throughout the task. A depiction of the quiet standing task is shown in Figure 1.

### 2.4. Data Acquisition

Surface electromyography was recorded bilaterally from the tibialis anterior (TA), medial gastrocnemius (MG), and soleus (SL) muscles while standing, using wireless EMG sensors (Trigno EMG sensors, Delsys, Boston, MA, USA). After thoroughly cleaning the skin with alcohol, the sensors were placed on each muscle in accordance with the SENIAM guidelines. To minimize the possibility of crosstalk between the electrodes, the EMG sensors were positioned at least 10 cm apart. The sampling rate was set to 2000 Hz, and the signals were filtered (band-pass filter 20–450 Hz) using a bioamplifier (Trigno Wireless System, Delsys, Boston, MA, USA). Concurrently, the ground reaction force was sampled at 1000 Hz during the task using two separate force plates (MG-1060, Anima, Tokyo, Japan). The force and EMG signals were synchronized using a trigger signal (Trigger Module; Delsys, Boston, MA, USA) and stored on a personal computer for subsequent offline analysis. 

### 2.5. Data Analysis

For the analysis, 30 s of data was used, excluding 5 seconds each at the beginning and end of the standing task, and was analyzed using a custom-written program in MATLAB (Version 2021b, MathWorks, MA, USA). For the EMG analysis, a fourth-order Butterworth high-pass filter (>20 Hz) was first applied, followed by full-wave rectification, as has been reported to enhance the firing rate information of motor neuron pools [27,28]. The auto-spectra (*P_xx_* and *P_yy_*) of the rectified EMG signals, denoted as *x* and *y*, respectively, along with the cross-spectra (*P_xy_)* derived from these identical signals, were subsequently computed using the discrete Fourier transform method and applied to non-overlapping segments, each comprising 2048 data points. This approach is consistent with the results of previous studies in this field [16,19]. The intermuscular coherence (|*C_xy_*(*f*)|^2^) was estimated using the following equation:

$${\left|C_{xy}\left(f\right)\right|}^{2}=\frac{{\left|P_{xy}\left(f\right)\right|}^{2}}{P_{xx}\left(f\right)・P_{yy}(f)}$$

where *f* denotes the frequency. Coherence was calculated for the unilateral synergistic muscle pair (SL–MG) and unilateral antagonist muscle pairs (TA–SL and TA–MG) on both the paretic and non-paretic limbs, together with the bilateral homonymous muscle pairs (SL–SL and MG–MG). Furthermore, a 95% confidence interval was calculated using the equation 1 − (0.05)^[1/(N−1)]^, where N represents the number of disjoint segments used for the coherence calculation. The intermuscular coherence was z-transformed and the area beyond the 95% confidence interval was calculated in the delta (0–5 Hz) and beta (15–35 Hz) bands. 

The COP data were first low-pass-filtered (<15 Hz) using a fourth-order zero-phase-lag Butterworth filter. The standard deviations of the COP displacement and COP sway velocity during the standing task were then calculated in the anteroposterior and mediolateral directions. In addition, the weight-bearing asymmetry was calculated from the average weight bearing during the task using the following formula:

$$\frac{\left(Non\;paretic\;limb-Paretic\;limb\right)}{\left(Non\;paretic\;limb+Paretic\;limb\right)}$$

### 2.6. Clinical Assessments

The participants underwent a clinical examination within a week before and after the experiment. The lower-extremity items of the Fugl–Meyer assessment (FMA-LE) [29] were used to quantify the motor impairment of the paretic lower limb. The Berg Balance Scale (BBS) was used as a clinical balance scale [30]. Gait performance was measured by determining a comfortable gait velocity along a 10 m walkway, with 3 m allocated for both acceleration and deceleration [31]. The Fall Efficacy Scale International (FES-I) was used as a psychological instrument assessing fall-related psychological factors [32]. Additionally, we assessed plantar flexor muscle spasticity using the modified Ashworth scale (MAS) [33]. Each clinical assessment was administered by the principal physical therapists entrusted with each participant’s care. It is pertinent to highlight that these assessment scales were verified to exhibit commendable interrater reliability [32,33,34].

### 2.7. Statistical Analysis

Paired *t*-tests were used to ascertain the differences in the coherence levels within the unilateral synergistic muscle pair and unilateral antagonist muscle pairs, specifically in the delta and beta frequency bands, between the paretic and non-paretic limbs. Furthermore, we computed the correlation between each intermuscular coherence value and parameters pertaining to the COP using Pearson’s correlation coefficients. We explored the relationship between the intermuscular coherence and clinical assessment scores, including FMA-LE, BBS, gait speed, FES-I, and MAS, employing either Pearson’s or Spearman’s rank correlation coefficients contingent upon the normal distribution of each variable. The statistical significance was set at *p* < 0.05.

## 3. Results

The characteristics of the patients with stroke who participated in this study are presented in Table 1. In general, the participants had mild to moderate hemiparalysis (FMA-LE: 23.1 ± 7.1) and also a mild to moderate degree of balance disturbance (BBS: 45.9 ± 9.1). The standard deviation of the COP displacement and COP velocity in the anteroposterior and mediolateral directions was 6.02 ± 1.81 mm, 3.75 ± 1.78 mm, 16.7 ± 5.62 mm/s, and 12.0 ± 6.19 mm/s, respectively, and the weight-bearing asymmetry was 0.22 ± 0.30.

**Figure 2:**
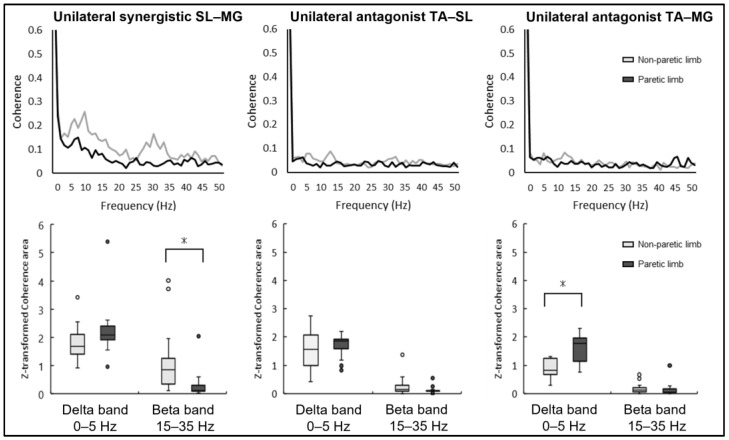
Representative intermuscular coherence of unilateral muscle pairs in paretic and non-paretic limbs. Small circles indicate outliners. The first row is the pooled coherence of each muscle pair; the second row is the area over the 95% confidence interval of the z-transformed coherence. * Significant difference (*p* < 0.05).

The results of the intermuscular coherence in the unilateral muscle pairs of the paretic and non-paretic limbs in each frequency band are shown in Figure 2. The unilateral synergistic muscle (SL–MG) coherence was significantly larger in the non-paretic limb compared to the paretic limb in the beta band (paretic limb: 0.30 ± 0.49, non-paretic limb: 1.20 ± 1.15, *p* = 0.017). Conversely, no statistically significant difference was noted between the paretic and non-paretic limbs in the beta band frequency concerning the unilateral antagonist muscle coherence of TA–SL (paretic limb: 0.13 ± 0.12, non-paretic limb: 0.26 ± 0.34, *p* = 0.06) and TA–MG (paretic limb: 0.15 ± 0.24, non-paretic limb: 0.19 ± 0.18, *p* = 0.49).

The coherence of the unilateral antagonist muscle pair (TA–MG) was significantly greater for the paretic limb compared to the non-paretic limb in the delta band (paretic limb: 1.60 ± 0.49, non-paretic limb: 0.90 ± 0.33, *p* < 0.01), while the other unilateral antagonist muscle pair (TA–SL) showed no significant difference in this frequency (paretic limb: 1.71 ± 0.40, non-paretic limb: 1.54 ± 0.72, *p* = 0.40). Furthermore, there was no significant difference between the paretic and non-paretic limbs in unilateral synergistic muscle (SL–MG) coherence in the delta band (paretic limb: 2.25 ± 0.93, non-paretic limb: 1.82 ± 0.60, *p* = 0.078).

A correlation analysis revealed a significant correlation between some muscle pairs with intermuscular coherence and COP parameters (Table 2). In the beta band frequency, unilateral synergistic muscle (SL–MG) coherence within the non-paretic limb was significantly correlated with the COP sway velocity in the anteroposterior direction (r = 0.518, *p* = 0.048). Moreover, the beta band coherence of the unilateral antagonist TA–SL pair was significantly correlated with the weight-bearing asymmetry in the non-paretic limb (r = −0.517, *p* = 0.048) and the standard deviation of the anteroposterior COP in the paretic limb (r = −0.533, *p* = 0.041). Furthermore, weight-bearing asymmetry was significantly correlated with the intermuscular coherence of the unilateral TA–MG pair (r = 0.608, *p* = 0.016) in the paretic limb, bilateral homonymous MG–MG (r = −0.602, *p* = 0.018), and SL–SL pair (r = −0.534, *p* = 0.041) in the delta band frequency.

The results of the correlation between the clinical assessments and intermuscular coherence are summarized in Table 3. The principal observation is that paretic antagonist TA–MG coherence and bilateral homonymous (SL–SL and MG–MG) coherence within the delta band exhibit a statistically significant correlation with scores in clinical assessments. 

## 4. Discussion

The current study revealed the existence of a higher degree of coherence in the unilateral synergistic muscle (SL–MG) of the non-paretic limb than in the paretic limb within the beta frequency band. Conversely, in the delta frequency band, the unilateral antagonist muscle coherence (TA–MG) was higher in the paretic limb than in the non-paretic limb. Furthermore, intermuscular coherence indices showing disparities between limbs (TA–MG and SL–MG) and coherence within the bilateral homonymous muscles (SL–SL and MG–MG) were correlated with the existing COP parameters and clinical assessments.

Intermuscular coherence within the beta frequency band reflects the descending neural drive of the corticospinal tract [11,35]. Coherence in the beta band frequency domain has been well documented, particularly in contexts involving relatively complex standing tasks that require intricate control in healthy individuals [18,19]. Conversely, stroke patients often encounter challenges even in tasks that are as fundamental as quiet standing. In fact, the participants in the present study demonstrated a greater extent of COP sway and COP velocity than previously reported for healthy older adults during a quiet standing task [18,19]. The heightened unilateral synergistic muscle (SL–MG) coherence observed within the non-paretic limb could potentially be attributed to escalated conscious modulation of the non-paretic plantar flexors by the unaffected cerebral cortex. The increased coherence of the non-paretic synergistic muscles appeared to be a compensatory activity in response to the difficulty of the standing task. This inference is supported by the significant and positive correlation detected between COP sway velocity and unilateral synergistic muscle (SL–MG) coherence in the non-paretic limb.

The unilateral antagonist TA–MG coherence in the delta band showed a significant positive correlation with the FES-I in the present study. This observation tentatively suggests that stroke patients with a greater fear of falling are more likely to have increased joint stability due to synchronous control between the antagonist muscles, thereby addressing their postural sway. In fact, a postural stiffening strategy involving the co-contraction of the ankle muscles has been suggested as a fear-related change in neuromuscular control when standing under fear-inducing conditions [36]. Moreover, augmented coherence between the paretic TA–MG was moderately to highly associated with worse clinical assessments scores, particularly affecting the FMA, BBS, and gait speed in the present study. Given that co-contraction of the ankle muscles serves as a compensatory strategy for addressing postural instability in stroke patients, this activation pattern is likely to be more pronounced in patients with greater paralysis severity. Nonetheless, it is worth noting that an excessive co-contraction among the antagonist muscles concurrently restricts the degrees of postural control and functional mobility of the COP, subsequently contributing to a reduction in overall functional balance and gait speed.

The bilateral homonymous coherence observed in the delta band between the SL–SL and MG–MG revealed a significant positive correlation with more favorable COP parameters, weight-bearing asymmetry, and clinical assessments. Throughout the quiet standing task, the bilateral homologous plantar flexors synergistically regulated the ankle joint torque [12,37,38], thereby substantively contributing to the maintenance of standing balance. Significant bilateral homonymous coherence has been consistently documented in numerous studies of healthy individuals [12,18,37,38]. However, contrary to the findings of the present study, previous studies conducted in healthy older adults have yielded contrasting results. Specifically, augmented bilateral homonymous muscle coherence was associated with inferior COP parameters [12,16,39]. This indicates that the impaired postural control observed in patients with stroke diverges from the mechanisms observed in healthy older adults. It is postulated that healthy older adults endeavor to adapt to amplified COP sway by executing compensatory augmentations in the descending control of the bilateral plantar flexors. In contrast, stroke patients may experience an elevated COP sway due to an inherent inability to heighten the descending control of the bilateral plantar flexors. Furthermore, greater coherence of the bilateral homonymous SL–SL and MG–MG in the delta frequency band demonstrated a significant positive correlation with more favorable clinical assessment scores (FMA, BBS, MAS, and gait speed). The increased bilateral homonymous coherence likely plays a role in maintaining a symmetrical standing posture. Stroke patients, on the other hand, exhibit a diminishment in this bilateral neuromuscular control, leading to compensatory mechanisms for postural stability, primarily relying on enhanced neural modulation within the non-paretic limb. Consequently, this predisposes them to adopt asymmetric standing, ultimately resulting in a compromised functional balance and gait ability. For individuals in the sub-acute stage of stroke, improving synchronous regulation of the bilateral plantar flexors, with reduced reliance on compensatory maneuvers within the non-paretic limb, holds significance in attaining an optimal standing balance and gait functionality.

One of the essential objectives in the rehabilitation of sub-acute stroke patients is a reduction in fall risk while concurrently enhancing their functional balance ability. Conventional clinical practice and research endeavors have predominantly focused on behavioral assessments, encompassing clinical assessment scales and postural sway assessments, with limited emphasis on evaluating the neuromuscular control characteristics. Nonetheless, given that stroke patients commonly exhibit asymmetrical standing control characteristics arising from hemiplegia, it becomes particularly imperative to assess these neuromuscular control attributes and their inherent asymmetry to enhance functional standing ability. This study has showed that sub-acute stroke patients demonstrate a compensatory augmentation in non-paretic plantar flexor activity from the contralateral lesion side. This compensation is accompanied by a concurrent rise in co-contraction among the paretic antagonist muscles. In contrast, synchronized control of the bilateral plantar flexor muscles diminishes. These asymmetries in the neuromuscular control characteristics represent potential mechanisms contributing to impaired standing balance. Therefore, it is plausible that this neuromuscular control pattern warrants assessment and targeted intervention during rehabilitation for sub-acute stroke patients. Consequently, the intermuscular coherence analysis employed in this study readily facilitates the estimation of neuromuscular control patterns and their correlated neural circuits, all derived from surface electromyography. This analytical approach holds substantial promise for further advancement in the domain of stroke rehabilitation.

## 5. Limitations

The present study had some limitations. First, because the study focused primarily on standing control under natural weight-bearing conditions, the between-limb differences observed in unilateral intermuscular coherence could have been influenced by weight-bearing asymmetry. However, it is pertinent to highlight that most previous studies investigating the COP sway during quiet standing in stroke patients have adopted conditions involving natural weight bearing. Furthermore, the correlation detected between the unilateral synergistic muscle coherence within the beta band and COP velocity rather than weight-bearing asymmetry suggests that between-limb differences in unilateral coherence are not solely driven by weight-bearing asymmetry. Second, there was no healthy control group; therefore, the reference values for intermuscular coherence, particularly for bilateral homonymous coherence, were lacking. Thus, the present study was designed to explore the associations between COP parameters, clinical measurements, and intermuscular coherence, and to verify the implications of intermuscular coherence in stroke patients. Furthermore, this study used a convenience sampling methodology without preceding the study with rigorous sample size estimation informed by power analysis, attributable to the pioneering nature of the present study within this research domain and its concomitant exploratory implications. The relatively modest sample size of this study primarily arises from the rigorous inclusion criteria. Numerous individuals with left-sided lesions, frequently linked to aphasia, did not meet these criteria and were consequently omitted. This exclusionary factor has the potential to curtail the applicability of our study’s outcomes. Finally, the present study was a cross-sectional investigation, and longitudinal changes in neuromuscular control are unknown. Future research is required to verify whether rehabilitation improves asymmetrical unilateral intermuscular coherence during standing tasks in patients with stroke. These inquiries would play a crucial role in elucidating whether engagement in rehabilitation strategies enhances the domains of neuromuscular control in these patients.

## 6. Conclusions

In conclusion, this study identified the differences in the unilateral intermuscular coherence patterns within the paretic and non-paretic limbs of patients with sub-acute stroke. Within the delta band, the unilateral antagonist muscle (TA–MG) coherence exhibited a greater magnitude within the paretic limb than in the non-paretic limb. Conversely, the beta band revealed heightened unilateral synergistic muscle (SL–MG) coherence within the non-paretic limb, in contrast to the paretic limb. These findings suggest that stroke patients adopt a postural strategy characterized by antagonistic muscle co-contraction and asymmetrical motor control engagement in the modulation of synergistic plantar flexors. Additionally, intermuscular coherence indices featuring between-limb differences (TA–MG and SL–MG) and bilateral homonymous muscle coherence were associated with COP parameters, thereby serving as valuable metrics for elucidating the neuromuscular dynamics underlying compromised postural stability in stroke patients.

## Figures and Tables

**Table 1:** Demographic and clinical characteristics of participants.

	Mean ± SD
Age (years)	55.7 ± 11.4
Height (m)	1.66 ± 0.06
Weight (kg)	65.3 ± 14.0
Sex (*n*): male/female	11/4
Lesion side (*n*): right/left	13/2
Time post-stroke (days)	55.3 ± 16.2
FMA-LE	23.1 ± 7.1
BBS	45.9 ± 9.1
MAS (*n*): 0/1/1 + /2/3/4	4/5/0/6/0/0
FES-I	33.8 ± 11.4
Gait speed (m/s)	0.71 ± 0.40
FAC (*n*): 1/2/3/4/5	1/5/3/2/4

Abbreviations: FMA-LE: Fugl-Meyer assessment of lower extremities, BBS: Berg Balance Scale, MAS: modified Ashworth scale of plantar flexors, FES-I: Fall Efficacy Scale International, FAC: Functional ambulation categories.

**Table 2:** Correlation coefficient (r) between intermuscular coherence and COP parameters.

		COP Parameters				
		SD AP	Velocity AP	SD ML	Velocity ML	WBA
**Delta band coherence**	**Paretic/Non-paretic**					
SL–MG	Paretic	0.24	0.49	0.14	0.15	−0.06
	Non-paretic	0.28	0.37	0.33	0.07	0.19
TA–SL	Paretic	−0.08	−0.35	−0.13	−0.26	0.18
	Non-paretic	−0.45	0.01	−0.21	−0.01	0.02
TA–MG	Paretic	0.15	0.33	0.39	0.42	0.61 *
	Non-paretic	−0.49	−0.12	−0.41	−0.14	0.02
SL–SL	Bilateral	−0.44	−0.51	−0.72 *	−0.71 *	−0.53 *
MG–MG	Bilateral	−0.28	−0.35	−0.40	−0.49	−0.60 *
**Beta band coherence**	**Paretic/Non-paretic**					
SL–MG	Paretic	−0.22	−0.24	−0.38	−0.21	−0.42
	Non-paretic	0.46	0.52 *	0.32	0.35	0.39
TA–SL	Paretic	−0.53 *	−0.51	−0.46	−0.34	−0.40
	Non-paretic	−0.34	−0.32	−0.40	−0.27	−0.52 *
TA–MG	Paretic	−0.46	−0.30	−0.40	−0.10	−0.30
	Non-paretic	−0.28	−0.23	−0.30	−0.14	−0.25
SL–SL	Bilateral	0.19	−0.23	−0.25	−0.13	−0.08
MG–MG	Bilateral	0.25	−0.29	−0.14	−0.36	−0.14

Abbreviations: AP: anteroposterior, ML: mediolateral, WBA: weight-bearing asymmetry, TA: tibialis anterior, SL: soleus, MG: medial gastrocnemius. * Significant correlation (*p* < 0.05).

**Table 3:** Correlation coefficient (r) between intermuscular coherence and clinical scales.

		FMA-LE	BBS	MAS	FES-I	Gait Speed
**Delta band coherence**	**Paretic/Non-paretic**					
SL–MG	Paretic	−0.19	−0.19	−0.08	0.36	−0.26
	Non-paretic	−0.33	−0.27	0.05	0.16	−0.15
TA–SL	Paretic	0.06	0.05	−0.22	−0.10	−0.05
	Non-paretic	−0.10	−0.01	0.26	0.16	−0.29
TA–MG	Paretic	−0.57 *	−0.67 *	**0.31**	0.77 *	−0.84 *
	Non-paretic	0.01	0.08	−0.01	0.15	−0.25
SL–SL	Bilateral	0.66 *	0.73 *	−0.64 *	**−0.35**	0.54 *
MG–MG	Bilateral	0.75 *	0.75 *	−0.62 *	**−0.27**	0.63 *
**Beta band coherence**	**Paretic/Non-paretic**					
SL–MG	Paretic	0.11	0.17	−0.32	−0.02	0.12
	Non-paretic	−0.15	−0.22	0.28	0.42	−0.17
TA–SL	Paretic	0.29	0.24	−0.42	−0.07	−0.03
	Non-paretic	0.37	0.38	−0.35	0.17	0.10
TA–MG	Paretic	0.29	0.28	−0.54 *	−0.09	0.18
	Non-paretic	−0.14	−0.10	0.09	0.33	−0.33
SL–SL	Bilateral	0.50	0.34	−0.41	0.08	0.34
MG–MG	Bilateral	0.36	0.51	−0.47	−0.60 *	0.60 *

Abbreviations: FMA-LE: Fugl-Meyer assessment in the lower extremities, BBS: Berg Balance Scale, MAS: modified Ashworth scale of plantar flexors, FES-I: Fall Efficacy Scale International, TA: tibialis anterior, SL: soleus, MG: medial gastrocnemius. * Significant correlation (*p* < 0.05).

## Data Availability

The data presented in this study are available on request from the corresponding author. The data are not publicly available due to the absence of participant consent for public sharing.
